# Optic tectum size correlates positively with eye size across, but not within, populations of Asiatic toads along altitudinal gradients

**DOI:** 10.1098/rsbl.2025.0487

**Published:** 2025-10-29

**Authors:** Zhongyi Yao, Jinzhong Fu

**Affiliations:** ^1^ Mountain Ecological Restoration and Biodiversity Conservation Key Laboratory of Sichuan Province, Chengdu Institute of Biology, Chinese Academy of Sciences, Chengdu 610213, China; ^2^ China-Croatia Belt and Road Joint Laboratory on Biodiversity and Ecosystem Services, Chengdu Institute of Biology, Chinese Academy of Sciences, Chengdu 610213, China; ^3^ Department of Integrative Biology, University of Guelph, Guelph, Ontario, Canada

**Keywords:** optic tectum size, eye size, Bufo gargarizans, trait co-variation, correlational selection

## Abstract

Co-variation with functionally integrated sensory structures contributes to the size evolution of brain regions. In this study, we investigated co-variation between sizes of the optic tectum and eye of Asiatic toads (*Bufo gargarizans*) along altitudinal gradients. The relative sizes of both the optic tectum and eye decrease significantly along with increases in altitude. Furthermore, optic tectum size is positively correlated with eye size among populations (*p* < 0.05, *R^2^* = 0.290), but not among individuals within each population, where natural selection occurs. The eye and optic tectum are likely subjected to parallel and independent local adaptation along altitudinal gradients that produce the correlative pattern; however, the pattern does not support functional integration and selection on their correlation. Eye size change may not have a significant impact on optic tectum evolution. Our study highlights the importance of cross-scale comparative analysis for inferring evolutionary processes.

## 1. Introduction

There is an ongoing debate over whether brain regions evolve in a coordinated fashion (the concerted brain evolution hypothesis) or follow independent evolutionary trajectories (the mosaic hypothesis). An increasing number of recent works across fishes, amphibians, and mammals have indicated that the relative size of different brain regions may indeed change independently [1–5]. These brain region size variations have often been attributed to correlated evolution with other sensory structures that are functionally integrated, as a consequence of brain region-specific selection (e.g. explicit sensory requirements). For example, the increased size of the cerebellum, but not the whole brain, in some teleost fishes is associated with the emergence of a novel electrosensory system [1].

Comparative analysis has played a key role in determining such trait correlation or integration. Most recent studies are between-species comparisons [1–4]. Nevertheless, trait correlation at different biological scales (i.e. species, population, individuals within population) implies very different evolutionary processes [6,7]. For example, trait correlation manifested within a population indicates integrated function and correlational selection. The traits may contribute to the same function and performance, and the trait combination itself is the target of selection (i.e. correlational selection), which occurs within populations [8,9]. On the other hand, trait correlation manifested at above population levels but not within population may suggest functional independence. The traits may not be genetically, structurally or functionally linked, even though they may correlate across an ecological gradient due to their parallel but independent local adaptation [6,10].

The optic tectum and eye are often assumed to be functionally linked within the visual perception system [11–13] and are subjected to correlational selection in several vertebrate groups (e.g. fishes, anurans and birds). The optic tectum is a functional specialist; it receives projections from the eyes, processes primary visual input and also engages in important bidirectional communication with the brainstem [14,15]. The potentially unified function and integration suggest that the two traits may strongly co-vary [13]. In fact, co-variation between eye size and the size of the optic tectum has been detected [14,16,17]. Furthermore, several ecological variables, including daytime light conditions for fishes and birds, lentic breeding environments for anurans and fishes and low habitat complexity for fishes, have been associated with small eye, small brain and in some cases small optic tectum [11,18–21]. The association has often been attributed to functional integration between the eye and optic tectum that large eyes have better sensitivity and/or resolution, and produce more information that requires a large optic tectum to process the information [18]. Nevertheless, the assumed functional integration between brain regions and sensory structures, and its impacts of brain evolution have rarely been explicitly tested.

Asiatic toads (*Bufo gargarizans*) along altitudinal gradients from the Hengduan Mountains present an excellent study system to test size co-variation between the optic tectum and eye and related evolutionary processes. The gradient system, as opposed to a binary system, provides continuous ecological variations, that likely lead to structured local adaptation and observable trends of trait variations. The area has strong altitudinal habitat zonation [22]; with increasing altitudes, shrub cover decreases and herb cover increases [23]. There is an overall reduction in habitat complexity with altitude. Also, the breeding habitat of these toads shifts from lotic to lentic water at high altitudes (our field observation, see electronic supplementary material, figure S1). Previous works have reported numerous trait variations of these toads along the altitudinal gradients, including reduced whole brain size, reduced optic tectum size and increased diurnal activities at high altitudes [5,24]. In anurans, the increased diurnal rhythm, lentic breeding water and reduced environmental complexity are known to be associated with reduced eye size [18]. Perhaps more importantly, this system is intraspecific, which allows both within- and among-population comparisons. Intraspecific comparison provides a more effective means of testing mechanistic hypotheses [25], and across-scale comparisons allow us to identify types of co-variation and to better infer processes [6,10,26,27]. For example, correlational selection on traits that work for a common function/performance usually leads to within-population co-variation but rarely results in the same relationships among populations or species [6,28].

In this study, we test co-variation between optic tectum size and eye size in Asiatic toads along altitudinal gradients, and explore the associated evolutionary processes. More specifically, we addressed two questions: (i) how eye size varies along altitudinal gradients and how it may co-vary with optic tectum size, and (ii) how their co-variation patterns, particularly co-variation within- and among populations, may inform us regarding brain region size variation and associated evolutionary processes. We predict that eye size would reduce along altitudes. High-altitudinal toads with increased diurnal activities and reduced habitat complexity likely have smaller eyes [18]. Our previous work has determined a decreased size of the optic tectum [5]. If the two traits have strong functional integration and correlational selection drive their co-variation, they would positively co-vary within populations.

## 2. Material and methods

### (a) Study sites and animals

Toads were breeding adults collected during their breeding seasons (2018 and 2019) near their breeding ponds in the Hengduan Mountains (electronic supplementary material, figure S2). All 15 sampling sites were along three altitudinal transects with the lowest site at 785 m.a.s.l. and the highest site at 3239 m. Sampling site 2.5 was excluded from subsequent analyses due to an insufficient sample size. Detailed information of sample sizes and sampling sites are provided in electronic supplementary material, tables S1 and figure S2. All animals were euthanized immediately at the collection sites. For euthanasia, toads were submerged in a 0.25% MS-222 solution for 10–25 min [5,29,30]. Death was confirmed when toads had their eyes closed and stopped moving. All procedures followed approved protocols (Chengdu Institute of Biology, no. 20171205).

### (b) Measurement of optic tectum and eye size

The estimated volume was used as the size of the optic tectum and measurement followed our previous work (see [5]). Briefly, whole brains were dissected out and the cranial nerves, pineal organ, meninx and pituitary glands were removed (but we kept the pituitary infundibulum). We took photographs of the brains from the top and side views, and measured the length (*L*), width (*W*) and height (*H*) of the right optic tectum and whole brain to the nearest 0.001 mm using ImageJ [31]. Each optic tectum was measured three times by the same investigator (Z.Y.), and we used the average value. The optic tectum closely approximates an ellipsoid, and its volume was estimated using a standard ellipsoid model *V* = (*L* × *H* × *W*) *π*/6. Then we doubled the calculated value as the total estimated size of optic tectum. Similarly, the whole brain was measured three times, and its volume was estimated using a modified ellipsoid model *V* = (*L* × *H* × *W*)*π*/(6 × 1.43) [32,33]. This model was modified for amphibian brains using a fluid displacement method [32].

The diameter of the transverse cornea was measured to represent the eye size [18,34]. We took side-view photographs of the right eyes, controlling head position and angle to ensure that the plane containing the eye was parallel to the camera sensor. Then we measured the diameter of the cornea along the major body axis to the nearest 0.001 mm using ImageJ. Each eye was measured three times by the same person (Z.Y.), and we used the average value. The cornea has a distinct border and is easy to measure, and has been shown to represent eye size well in anurans [18]. The snout–vent length (SVL) was used to represent body size and was similarly measured from photographs that were taken from the top view.

Damaged organs were excluded from measurement. Finally, data from a total of 324 specimens from 14 sites (populations) were gathered (electronic supplementary material, table S1).

### (c) Statistical analysis

We first confirmed linear allometry relationships within each population between eye size and body size (SVL) and between optic tectum size and whole brain size (all four measurements were log transformed). Residuals versus fitted plots showed that data points are randomly scattered around zero with no obvious curve or funnel shape (electronic supplementary material, figures S3 and S4). To obtain relative eye size (to body size) and relative optic tectum size (to whole brain size), we performed allometric correction by population ( = sampling site) using the Thorpe method implemented in *R* package *GroupStruct* version 0.1.0 [35,36]. SVL was used as the correcting covariable for eye size, while whole brain volume was used as the correcting covariable for the optic tectum. The allometric correction follows the equation:


$$X_{adj}=\log\left(X\right)-b\left[\log\left(BL\right)-\log\left(BL_{mean}\right)\right],$$

where $X_{adj}$ is the corrected value for the trait (e.g. eye size); X is raw trait value; b is the regression coefficient for $\log\left(X\right)$ against $\log\left(BL\right)$; BL is the measured body size SVL or whole brain volume; ${BL}_{mean}$ is the mean SVL or brain volume within the population. Then we standardized the sizes to a mean of zero and a variance of one.

To explore the size variation of the eye and optic tectum along the altitudinal gradient at the among-population level, we used general linear models (GLMs). In the models, mean values of relative eye size or optic tectum size in each population were set as dependent variables, while altitude was set as the independent variable.

We also used GLMs to test correlation between optic tectum size and eye size. In the models, relative optic tectum size was the dependent variable and relative eye size was the independent variable. For within-population comparison, we used individual as data point and examined the model for each population. For the among-populations comparison, we used the population mean value of relative eye size and optic tectum size as the data point.

Model assumptions, including normality of residuals and homoscedasticity, were checked, and there was no significant violation of these assumptions. All modelling analysis was conducted using *R* [37] and all figures were generated using R package *ggplot2* [38].

## 3. Results

**Figure 1.**
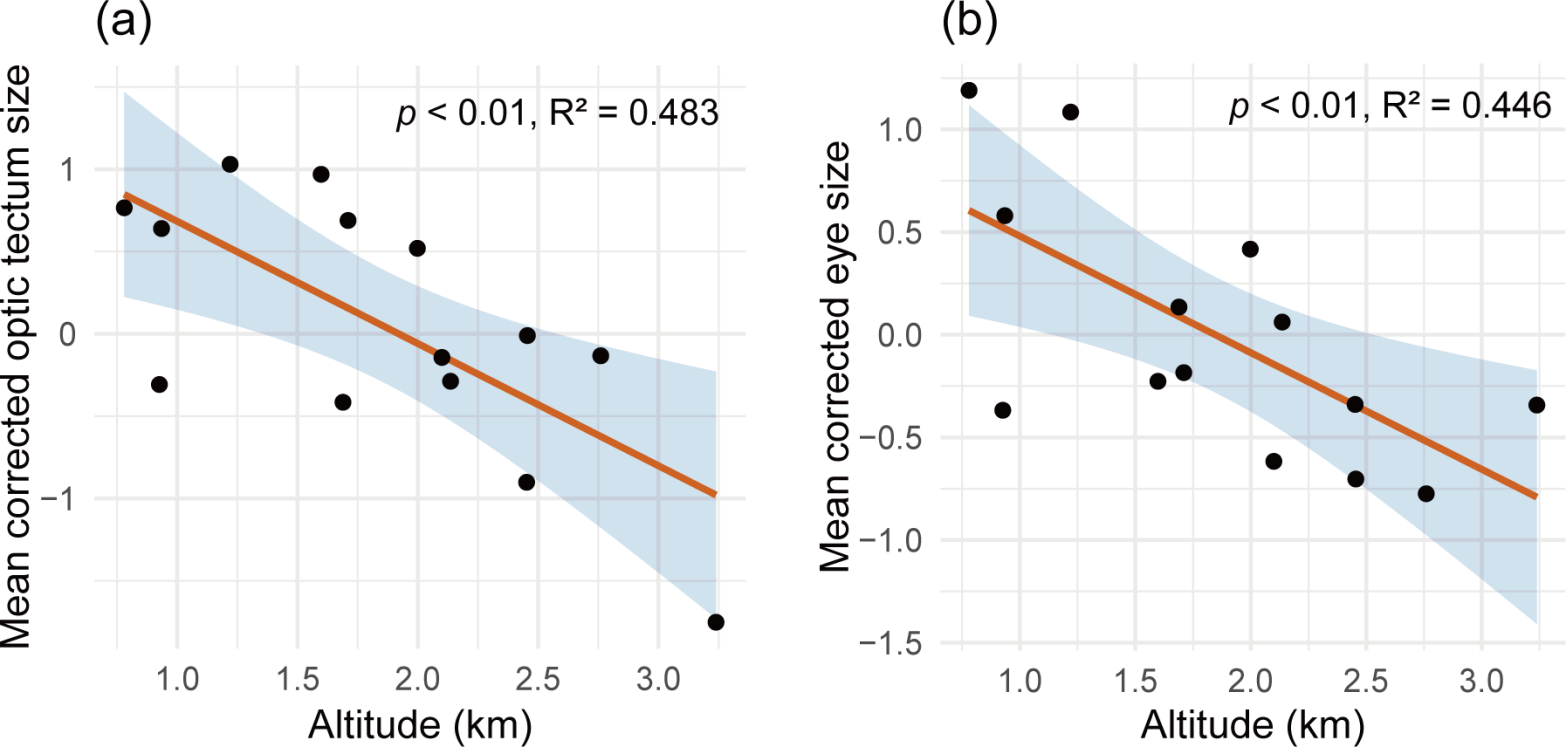
Size variations of the optic tectum (a) and eye (b) along altitudinal gradients. The size of the optic tectum is corrected against whole brain size, and eye size is corrected against SVL. Each data point is a population. The lines are GLM predictions.

Both eye size and optic tectum size displayed a significant negative correlation with altitude (GLM: eye, estimate ± s.e. = − 0.568 ± 0.183, *p* < 0.01; optic tectum, estimate ± s.e. = − 0.742 ± 0.222, *p* < 0.01; figure 1a,b and electronic supplementary material, table S2). Figure 1a shows the populations mean values of optic tectum size and the model-predicted trend line with altitudes, and figure 1b shows the populations mean values of eye size and the model-predicted trend line with altitudes.

**Figure 2.**
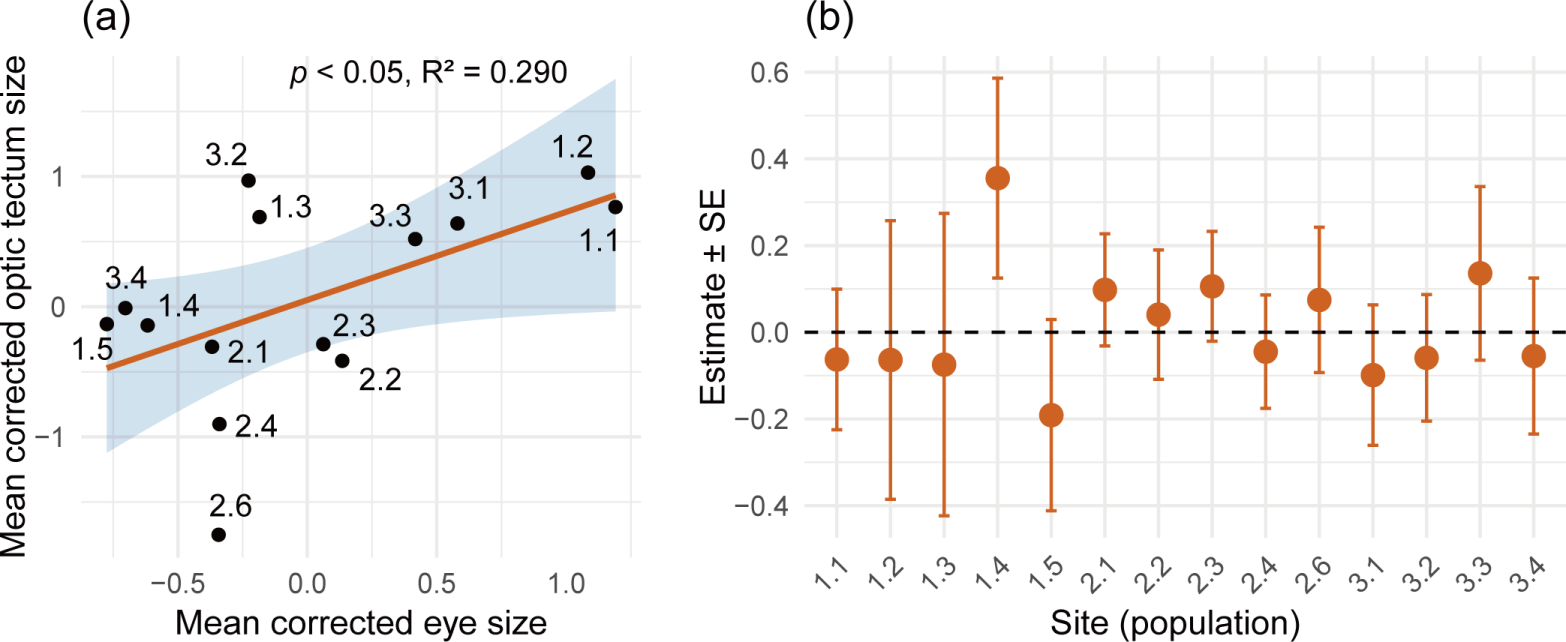
Correlation between relative optic tectum size and relative eye size among populations (a) and within populations (b). Each data point is a population and estimate (= slope) values are from GLM. None of the within-population correlation is significant (*p* > 0.05). Site 2.5 does not have an estimate due to its small sample size.

A significant positive correlation between eye size and optic tectum size was detected at the among-populations level (GLM: estimate ± s.e. = 0.676 ± 0.306, *p* < 0.05; figure 2a and electronic supplementary material, table S3). At the within-population level, however, GLMs indicated no significant correlation between eye size and optic tectum size in any of the populations (electronic supplementary material, table S4). Moreover, the coefficients for the effect of eye size on optic tectum size were inconsistent with both positive and negative values (figure 2b).

## 4. Discussion

The lack of within-population co-variation suggests that there is no correlational selection and the functional integration between the two traits is not the target of selection. Both eye size and optic tectum size respond independently to selection, and are adapted to specific ecological conditions of the local sites along the altitudinal gradient.

The eye size variation pattern of these toads is consistent with local adaptation along the altitudinal gradients. Eye size is closely related to visual acuity, which represents a key capacity for animal predators, and is often under selective pressure across many vertebrates [12,19,39–42]. There are several potential causes that may lead to a reduced eye size at high altitudes. While toads at low altitudes are dominantly nocturnal, high-altitude toads are more diurnal [24], and nocturnal activity has long been associated with large eyes in vertebrates [18]. The increased UV radiation at high altitudes may also favour small eyes, which likely reduces UV damage [43]. Additionally, low altitudes have heterogeneous habitats and an overall high environmental complexity, and large eye size is often favoured in complex environments [11,42]. Furthermore, toads are heavily reliant on specialized visual cues for predation [44,45], and variations in prey type and prey availability along altitude may also contribute to eye size variation. A toad population at a specific altitude likely has a local optimal eye size and a variation range around the value.

The reduced optic tectum size along with a reduced whole brain size among high-altitude toads was previously reported and has been attributed to trade-off with other energetically expensive organs [5,30]. Toads at different altitudes have a similar standard metabolic rate, when measured at the same temperature [24]. With low temperature and short activity season, high-altitude toads have low metabolic rates and experience energetic constraints that are not observed at middle and low altitudes [30]. A small brain is likely favoured at high altitudes. It has also been noticed that the relative size (to the whole brain) of the telencephalon is enlarged among high-altitude toads [5]. The accelerated reduction of optic tectum, compared with the whole brain, is likely a compensation for the enlarged telencephalon, at least in part.

Our data appear to be in line with the mosaic hypothesis that brain regions may evolve independently; however, our data do not support one of the primary mechanisms of the hypothesis, that independent variations of brain regions are consequences of different sensory requirements [1,46]. We did not detect co-variation between eye size and optic tectum size within any populations. Our sample sizes are reasonably large (*n* > 20 most populations; electronic supplementary material, table S4). If the functional integration between the two traits is strong, the trait combination would be the target of a correlational selection, which occurs within populations [6,30]. In such a case, we would expect significant co-variation between the two traits at the within-population level. Although eye size theoretically has a significant effect on visual sensitivity and resolution, which should correlate with visual sensory processing by the optic tectum, the association may not be strong enough to create a correlational selection that has a detectable impact on the size evolution of the optic tectum. Alternatively, eye size may not be a good representation of sensory requirements.

The observed co-variation at the among-population level can be best explained by parallel and independent local adaptation (or co-specialization [6]) of the eye and optic tectum. At each site, eye size responds to an altitude-specific selection regime and changes to the local optimal value. Random variations and migration maintain trait variation within each population but near its optimal value. The optic tectum size varies in a similar way but independently [30]. Since both traits are responding to the same altitudinal gradient in a linear fashion, the parallel local adaptation leads to correlation of trait means among local populations. A similar scenario has been observed in an amphibious fish, *Kryptolebias marmoratus*, where trade-off between two functionally independent traits were observed between, but not within, populations [10].

Our understanding of brain evolution will continue to benefit from comparative analysis. This study highlights the importance of cross-scale comparative analyses for accurately inferring evolutionary processes [6,7]. Co-variation with other traits, particularly sensory structures, will undoubtedly impact the brain region variations; however, the assumed functional integration and how correlational selection may contribute to their evolution should be explicitly tested.

## Data Availability

The raw data and analysis code supporting this study is available in ScienceDB at https://www.scidb.cn/en [47]. Supplementary material is available online [48].
